# Transfemoral TAVI in a High-Risk Patient with Porcelain Aorta and Severe Subrenal Abdominal Aortic Stenosis: A Case Report

**DOI:** 10.3390/jcdd12100396

**Published:** 2025-10-07

**Authors:** Anees Al Jabri, Marcello Ravani, Giuseppe Trianni, Tommaso Gasbarri, Marta Casula, Sergio Berti

**Affiliations:** 1 Department of Cardiology, G. Pasquinucci Heart Hospital, Fondazione Monasterio, 54100 Massa, Italy; 2 Department of Adult Cardiac Surgery, G. Pasquinucci Heart Hospital, Fondazione CNR Toscana Gabriele Monasterio, 54100 Massa, Italy; 3 Clinical Experimental Cardiology, Clinical and Interventional Cardiology, University of Sassari, 07100 Sassari, Italy

**Keywords:** aortic valvular stenosis, TAVI, porcelain aorta, transfemoral access, IVL

## Abstract

Aortic stenosis (AS) is a common degenerative valvular disease in elderly patients, causing obstruction of left ventricular outflow and presenting with symptoms such as angina, syncope, and heart failure. Although surgical aortic valve replacement (SAVR) remains the gold standard, its high perioperative risk in frail patients has led to the adoption of transcatheter aortic valve implantation (TAVI) as a less invasive and effective alternative. The transfemoral (TF) access route is generally preferred, but severe peripheral arterial disease may limit its feasibility. We report the case of a 71-year-old woman with critical AS complicated by multiple comorbidities, including extensive vascular calcifications, a porcelain aorta, and significant subrenal abdominal aortic stenosis. Multimodal imaging, including computed tomography, was essential for procedural planning, revealing complex iliofemoral anatomy unsuitable for conventional device passage without intervention. Intravascular lithotripsy (IVL) was used to disrupt calcific plaques and facilitate safe vascular access. The TAVI procedure was successfully performed under local anesthesia via TF access using a 65 cm GORE® DRYSEAL Flex Introducer Sheath (W. L. Gore & Associates, Flagstaff, AZ, USA) (18-Fr). After balloon valvuloplasty performed over a SAFARI2™ Pre-Shaped TAVI Guidewire, Extra Small (Boston Scientific, Marlborough, MA, USA) Curve in the left ventricle, a self-expanding Medtronic Evolut™ FX 26 (Medtronic, Minneapolis, MN, USA)mm transcatheter valve was implanted. Postoperative imaging confirmed optimal valve function and vascular integrity without complications. This case highlights the role of IVL as an innovative adjunctive technique enabling TF-TAVI in patients with challenging vascular anatomy, thereby expanding treatment options for high-risk individuals with severe AS.

## 1. Introduction

Aortic stenosis (AS) is a common valvular condition among the aging population, characterized by progressive degenerative changes that narrow the aortic valve orifice, resulting in left ventricular (LV) outflow obstruction [1]. Clinically, AS presents with symptoms such as chest pain, exertional syncope, and exertional dyspnea. While surgical aortic valve replacement (SAVR) has traditionally been the standard treatment for severe AS, the perioperative mortality risk—especially in elderly patients—has led to the emergence of transcatheter aortic valve implantation (TAVI) as a preferred, less invasive alternative that offers good outcomes [2]. Although various access routes are available for TAVI, the transfemoral (TF) approach remains the preferred method due to its high efficacy [3].

## 2. Case Presentation

In January 2025, a 71-year-old woman was admitted to the cardiology clinic for acute pulmonary edema (New York Heart Association (NYHA) class IV). Upon examination, her blood pressure was 140/70 mmHg, and her heart rate was 60 beats per minute. Cardiac auscultation revealed a loud ejection murmur indicative of AS, radiating to the carotid arteries. A 2D echocardiogram confirmed severe AS. Additional comorbidities included hypertension, former smoking, chronic obstructive pulmonary disease, rheumatoid arthritis, and chronic multi-infarct ischemic vascular encephalopathy. The patient also exhibited significant multidistrict arterial disease, including left carotid stenosis, a porcelain aorta, and subrenal abdominal aortic stenosis. Her home therapy consisted of Clopidogrel 75 mg daily, Atorvastatin 40 mg, and a loop diuretic.

**Figure 1:**
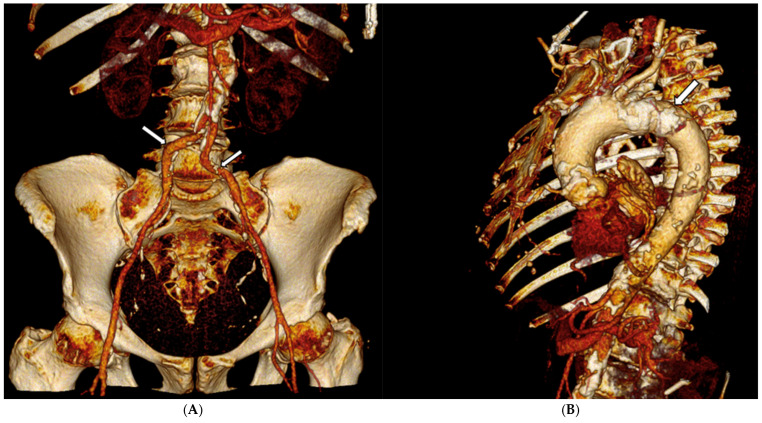
(**A**) Volumetric 3D CT angiographic reconstruction of the iliofemoral axis (arrows) demonstrating extensive calcified atherosclerotic disease; (**B**) Sagittal volumetric 3D CT angiographic reconstruction of the thoracoabdominal aorta reveals extensive calcified atherosclerotic disease indicative of porcelain aorta (arrow).

**Figure 2:**
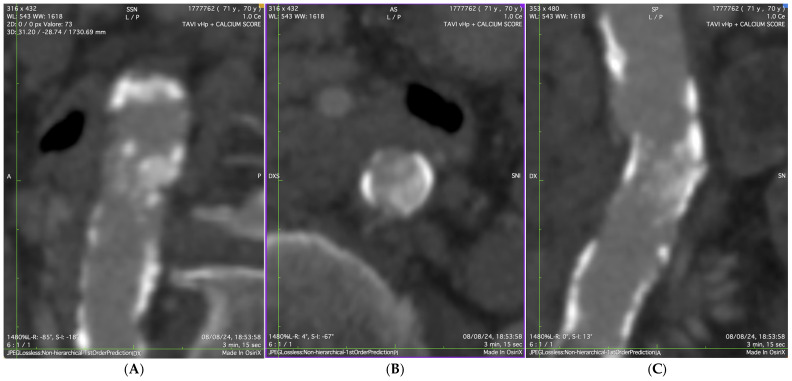
Multiplanar CT reconstructions of the subrenal abdominal aorta showing extensive calcification. (**A**) coronal view; (**B**) axial (transverse) section demonstrating concentric calcific stenosis; (**C**) sagittal view.

**Figure 3:**
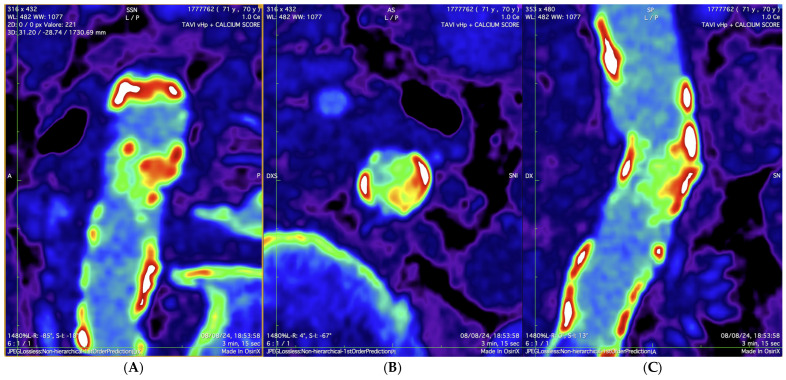
Color-coded calcium scoring CT reconstruction illustrating the detailed distribution and extent of calcification within the subrenal abdominal aorta, where red-to-white areas indicate dense calcified plaques and blue-to-violet tones represent surrounding soft tissues. (**A**) coronal view; (**B**): axial (transverse) view; (**C**) sagittal view.

A 12-lead electrocardiogram showed sinus rhythm, normal atrioventricular conduction, and ventricular repolarization abnormalities due to severe hypertrophy. Transthoracic echocardiography revealed a severe AS (maximum velocity = 436 cm/s, Doppler velocity index = 0.20, aortic valve area (AVA) = 0.62 cm^2^, AVA index = 0.40 cm^2^/m^2^), with normal LV ejection fraction (EF 60%), severe ventricular hypertrophy, moderate left atrial dilation, and moderate mitral stenosis. A computed tomography (CT) scan of the thoracic and abdominal aorta, along with lower extremities, was performed to assess the feasibility of TAVI. The CT scan revealed a diffusely atheromatous thoraco-abdominal aorta (Figure 1A,B), with severe subrenal abdominal aortic stenosis (minimal luminal diameter: 6.1 mm) (Figure 2 and Figure 3). The iliac arteries were also atheromatous but without significant stenotic or dilatative pathology: common (6 × 6.3 mm diameter on the right and 6.55 × 6.78 mm diameter on the left) and external iliac arteries (5 × 5.1 mm diameter on the right and 5.1 × 5.2 mm diameter on the left). The right common femoral artery (RCFA) showed no significant calcification, with a maximal luminal diameter of 5.8 mm and a minimal luminal diameter of 5.8 mm. A coronary angiography was conducted to assess the presence of coronary artery disease (CAD) and obtain hemodynamic measurements, showing diffuse CAD without angiographically significant stenoses. The preoperative risk was assessed with the Society of Thoracic Surgeons (STS) score for short-term mortality (5%) and the Euroscore II (6%). The Heart Team evaluated the patient and determined that the mortality risk for SAVR would be high; therefore, they recommended TAVI via TF access, ruling out other approaches such as transapical, transaortic, transcarotid, or transsubclavian. The TAVI procedure was planned to follow adequate preparation of the subrenal abdominal aorta using intravascular lithotripsy (IVL). The presence of extensive atherosclerotic disease involving multiple vascular districts, together with the center’s substantial expertise in the TF approach and the use of IVL, were decisive factors in supporting this strategy.

The procedure was performed under local anesthesia. Access to the RCFA was achieved under ultrasound guidance, with pre-positioning of Perclose™ ProStyle™ Suture-Mediated Closure and Repair (SMCR) System (Abbott Vascular, Santa Clara, CA, USA). A second access point was established via the right radial artery with 6F sheaths, through which a 6F pig-tail catheter was advanced into the right coronary cusp. A peripheral workhorse guide wire was then advanced into the abdominal aorta, and ten cycles of IVL were performed using Shockwave™ Intravascular Lithotripsy (IVL) balloon catheter (Shockwave Medical, Santa Clara, CA, USA) (L6 12.0 × 30 mm, 30 pulses per 10 cycles for a total of 300 pulses) at the level of the subrenal abdominal aorta (Video S1). Angiographic control showed an improvement in lumen diameter, preserved anterograde flow, and no signs of dissection flap.

Considering the porcelain aorta, especially the diffuse calcification of the aortic arch, a 65 cm GORE® DRYSEAL Flex Introducer Sheath 18-Fr was advanced up to the aortic arch. (Video S2). Subsequently, after placing a super-stiff SAFARI XS guidewire into the LV, balloon aortic valvuloplasty was performed by advancing VACS II 20 × 40 mm balloon (Osypka Medical, Rheinfelden, Germany) through the RCFA and inflating it across the aortic valve during rapid ventricular pacing at 180 beats per minute to relieve the stenosis. Rapid ventricular pacing temporarily reduced cardiac output, facilitating balloon inflation across the valve.

Then, through the super-stiff SAFARI XS guidewire in the LV, an Evolut FX 26 mm (Medtronic) prosthesis was implanted while pacing at 140 bpm. Control aortography confirmed correct positioning of the prosthesis, with the presence of a mild paravalvular leak (Video S3). The final angiographic check through the introducer demonstrated maintained anterograde flow with no evidence of an intimal flap in the subrenal abdominal aorta (Video S4)**.**

Hemostasis of the RCFA access was achieved by suturing with the Perclose™ ProStyle™ SMCR and an Angio-Seal™ Hemostasis System 8Fr (Terumo Medical Corporation, Somerset, NJ, USA), along with a cutaneous stitch. Echocardiographic control documented the patency of the right iliofemoral arterial axis.

## 3. Discussion

Transcatheter aortic valve implantation (TAVI) is a well-established therapeutic intervention for severe aortic stenosis in patients classified as intermediate to high risk. While the TF approach is preferred, peripheral artery disease can complicate access. The Valve Academic Research Consortium (VARC) reports that the incidence of major vascular complications (VCs) associated with the TF approach ranges from 5.0% to 23.3% [4]. These complications predominantly affect the iliofemoral arterial segment, underscoring the need for thorough preprocedural evaluation.

The literature on the correlation between valve type and the occurrence of vascular complications is mixed. A study by Demirci et al. [5] which utilized various next-generation transcatheter heart valves, found no significant difference in VCs based on valve type. Conversely, Koren et al. [6] reported a higher incidence of VCs in self-expanding valves compared to balloon-expandable valves.

Preprocedural planning using CT scan has become standard for pre-TAVI assessment, providing detailed three-dimensional visualization of aorto-iliofemoral anatomy. The minimum luminal diameter required for femoral access varies by valve type; for Medtronic valves, a minimum diameter of 5 mm is specified for the Evolut, Evolut Pro, and Evolut PRO+ valves (Medtronic, Minneapolis, MN, USA). In patients with complex vascular anatomy and severe calcification, IVL has recently emerged as an effective strategy to facilitate TF access. By generating pulsatile sonic pressure waves, IVL fractures medial calcium while preserving soft vascular structures, thereby allowing controlled vessel expansion at low inflation pressures.

The Disrupt PAD studies highlight the efficacy and safety of IVL, demonstrating lower rates of complications such as arterial dissection, residual stenosis, and stent placement requirements compared to routine angioplasty [7]. This success is attributed to lower maximum inflation pressures (4 atm) and the atraumatic fragmentation of medial calcium. Clinical experience with IVL in the TAVI setting is progressively expanding. In the largest multicenter European registry, Nardi et al. demonstrated that IVL-assisted transfemoral TAVI achieved 100% success in valve delivery and a 98.2% overall procedural success, with a low rate of major vascular complications, thereby confirming its safety and efficacy in patients with severely calcified iliofemoral disease [8]. More recently, Linder et al. compared IVL-TAVI with transaxillary access in patients with hostile iliofemoral anatomy and reported a higher technical success (93.3% vs. 81.8%) and significantly fewer safety events at 30 days (10.0% vs. 31.8%, *p* = 0.047) in the IVL group, supporting IVL as a strategy that both expands transfemoral indications and mitigates risks associated with alternativoke access routes [9]. In our case, IVL was crucial to safely dilate the subrenal abdominal aorta, enabling the advancement of a large-bore delivery system despite extensive calcific disease. Importantly, the most distinctive feature was the use of the largest available IVL balloon (L6, 12 mm) in the subrenal abdominal aorta, rather than being confined to the iliac or femoral arteries as commonly reported. This case underscores the potential of IVL not only in facilitating device passage through peripheral access vessels but also in the abdominal aorta, where extensive calcification and severe stenosis would otherwise preclude transfemoral TAVI.

## 4. Conclusions

Aortic stenosis is the most common acquired valvular heart disease in the elderly, often complicated by multiple comorbidities and challenging vascular anatomies. This case illustrates the feasibility of transfemoral TAVI in the presence of porcelain aorta and severe subrenal abdominal aortic stenosis, made possible through IVL. Importantly, IVL proved not only useful in facilitating device passage through femoral and iliac arteries, but also feasible and effective in the abdominal aorta, thereby further broadening the therapeutic horizon of this technology. Careful pre-procedural planning combined with IVL enabled the safe advancement of a large-bore delivery system, avoiding the need for alternative access routes. These findings reinforce the growing role of IVL in expanding transfemoral TAVI indications and providing minimally invasive solutions for high-risk patients with hostile iliofemoral anatomy.

## Data Availability

The data presented in this study are available on request from the corresponding author due to privacy.

## Supplementary Materials

The following supporting information can be downloaded at: https://www.mdpi.com/article/10.3390/jcdd12100396/s1, Video S1: Angiographic view showing intravascular lithotripsy using a Shockwave balloon at the level of the subrenal abdominal aorta; Video S2: Angiographic view showing an 18-Fr, 65 cm DrySeal Flex Gore valved introducer advanced up to the aortic arch; Video S3: Control aortography confirmed correct prosthesis positioning, revealing a mild paravalvular leak; Video S4: Final angiographic assessment through the introducer showing preserved antegrade flow without evidence of dissection or intimal flap at the level of the subrenal abdominal aorta.

## Abbreviations

The following abbreviations are used in this manuscript: AS = Aortic Stenosis; LV = Left Ventricle; SAVR = Surgical Aortic Valve Replacement; TAVI = Transcatheter Aortic Valve Implantation; TF = Transfemoral; NYHA = New York Heart Association; 2D = Two-Dimensional (echocardiogram); COPD = Chronic Obstructive Pulmonary Disease; RCFA = Right Common Femoral Artery; AVA = Aortic Valve Area; EF = Ejection Fraction; CT = Computed Tomography; CAD = Coronary Artery Disease; STS = Society of Thoracic Surgeons; IVL = Intravascular Lithotripsy; SMCR = Suture-Mediated Closure and Repair; Fr = French (catheter size unit); bpm = beats per minute; VARC = Valve Academic Research Consortium; VCs = Vascular Complication.
